# Trends and Disparities in Cardiovascular Disease in US Adults with Metabolic Dysfunction-Associated Steatotic Liver Disease

**DOI:** 10.3390/biomedicines13040956

**Published:** 2025-04-13

**Authors:** Yanbing Zhang, Xinge Zhang, Chuiguo Huang, Lei Zhu

**Affiliations:** 1 Department of Hepatobiliary Surgery, Renmin Hospital of Wuhan University, Wuhan 430060, China; zhangyanbing@whu.edu.cn; 2 Department of Medicine and Therapeutics, The Chinese University of Hong Kong, Hong Kong SAR, China; xinge@link.cuhk.edu.hk (X.Z.); chuiguohuang@cuhk.edu.hk (C.H.)

**Keywords:** cardiovascular disease, metabolic dysfunction-associated steatotic liver disease, prevalence, trends, disparity

## Abstract

**Background/Objectives**: Recently, the term metabolic dysfunction-associated steatotic liver disease (MASLD) has replaced non-alcoholic fatty liver disease (NAFLD). Through analysis of the trends and disparities regarding cardiovascular disease (CVD) among individuals with MASLD, identifying the leading cause of death in this population is crucial. **Methods**: We conducted a cross-sectional analysis of National Health and Nutrition Examination Survey (NHANES) III (1988–1994) and NHANES 2017–2020 data. MASLD was identified by using clinical profiles and liver ultrasonography to exclude other liver diseases. We estimated the prevalence of CVD among individuals with MASLD and calculated the prevalence ratios for those with and without MASLD. **Results**: In 2017–2020, MASLD affected 31.2% or 61.9 million US adults, and 17.0% (95% confidence interval: 13.7–20.3%) of these individuals had CVD. The absolute CVD prevalence in individuals with MASLD doubled from that in the NHANES III cohort, which was 8.7% (6.4%, 10.9%). These increases were especially notable among older adults, non-Hispanic whites, and those with higher education and income. Individuals with MASLD had a higher prevalence of total CVD than those without MASLD, even after adjusting for socioeconomic and metabolic factors. These differences were more pronounced in younger age groups. **Conclusions**: This study revealed a doubled 30-year trend in CVD prevalence among adults with MASLD in the US. Sociodemographic disparities emphasize the need for tailored screening, prevention, and policy measures to address gaps and promote cardiovascular health in this population.

## 1. Introduction

Non-alcoholic fatty liver disease (NAFLD) is a pathological condition characterized by the accumulation of fat in >5% of hepatocytes, occurring in the absence of secondary chronic liver injury (e.g., excessive alcohol consumption, chronic viral hepatitis, or drug-induced liver injury) [1]. Globally, an estimated 32.4% of adults have NAFLD [2]. In the US, the prevalence of NAFLD has steadily increased over the decades, reaching 31.9% in the years 2013–2016 [3]. NAFLD significantly increases the risk of liver-related complications and overall mortality, thereby posing a substantial health burden [4]. Despite alarming data on the prevalence and impact of NAFLD, awareness remains low among the general public and healthcare practitioners [5]. This low awareness is partly due to the names “non-alcoholic” and “fatty”. The term “non-alcoholic” defines the disease by way of exclusion, neglecting its strong link with metabolic abnormalities, while “fatty” is seen as stigmatizing, potentially hindering diagnosis and discussion [5]. Recognizing these issues, a global multisociety committee of experts convened to adopt new terminology and definitions to increase awareness and reduce stigma [5]. This new nomenclature introduces “steatotic liver disease” (SLD) as an umbrella term, under which NAFLD has been proposed to be replaced by “metabolic dysfunction-associated steatotic liver disease” (MASLD). Unlike NAFLD, MASLD requires the presence of at least one cardiovascular risk factor [5]. This redefinition necessitates a reassessment of the disease burden to accurately capture its prevalence and associated health outcomes.

Cardiovascular disease (CVD) is the leading cause of death in patients with NAFLD [6,7,8,9]; however, the prevalence and trends of CVD among the US population with MASLD remain unclear. In addition, disparities in the CVD burden among US patients with MASLD based on sociodemographic characteristics have not been previously reported, representing a critical gap in knowledge that is essential for the development of targeted public health campaigns and informed policy decisions aimed at reducing health inequities.

To address these gaps, we conducted a comprehensive evaluation of the prevalence and trends of CVD among the US population with MASLD from 1988 to 2020, examining both overall patterns and variations based on sociodemographic characteristics.

## 2. Materials and Methods

### 2.1. Data Source and Population

Data from the National Health and Nutrition Examination Survey (NHANES) III and NHANES 2017–2020 were utilized. NHANES III, conducted between 1988 and 1994, was a nationally representative survey designed to monitor the health and nutritional status of the civilian, non-institutionalized US population. Details of the study design, protocol, and data collection are described elsewhere [10]. Extensive data were gathered through interviews, physical examinations, and laboratory tests, including gallbladder ultrasound video images, which are necessary for diagnosing hepatic steatosis. Since 1999, the NHANES has been conducted every two years [11] through household interviews and standardized medical examinations, including blood sample collections performed in mobile examination centers [12]. Among these waves, NHANES 2017–2020 (a combined cycle from 2017 to March 2020 due to the COVID-19 pandemic) was chosen because it is the only one with available data on liver ultrasound-based transient elastography [13]. The NHANES received approval from the National Center for Health Statistics Research Ethics Review Board, and the participants provided written informed consent. This study adhered to the Strengthening the Reporting of Observational Studies in Epidemiology (STROBE) Reporting Guidelines.

**Figure 1:**
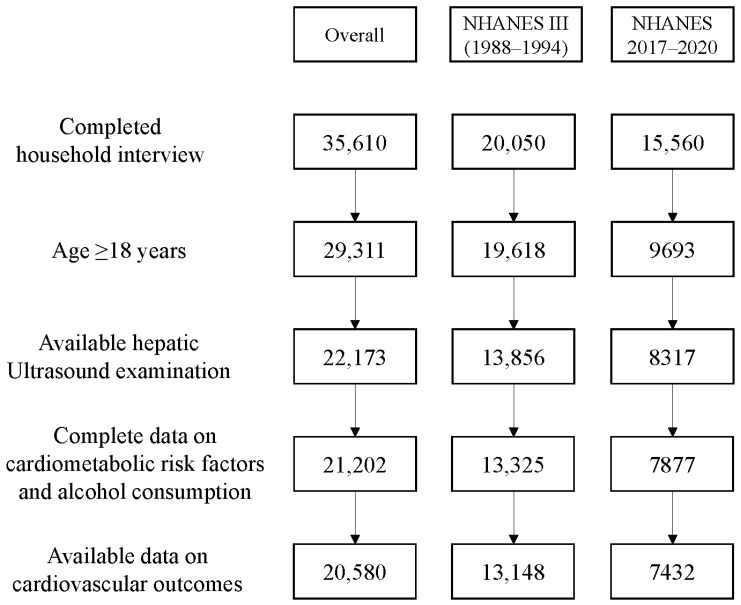
Flowchart of the inclusion process.

Only individuals aged ≥18 years who underwent a hepatic ultrasound examination were included. Individuals for whom information was lacking on cardiovascular risk factors, alcohol consumption, and/or cardiovascular outcomes were excluded. In total, 20,580 individuals were included in the final analysis. A flowchart of the exclusion process is shown in Figure 1.

### 2.2. Assessment of MASLD

MASLD was defined as the presence of SLD along with one or more cardiometabolic criteria and the absence of other discernible causes, including alcohol-related liver disease (ALD), metabolic alcohol-related liver disease (MetALD), and other etiologies [5]. SLD was defined as any degree of ultrasonographic hepatic steatosis or a median controlled attenuation parameter score cutoff of ≥248 decibels per meter [14]. The cardiometabolic risk factors included a BMI of ≥25 kg/m^2^, a waist circumference of ≥102 cm for men or ≥88 cm for women, a fasting serum glucose level of ≥100 mg/dL or hemoglobin A1c level of ≥5.7%, type 2 diabetes, a blood pressure of ≥130/85 mmHg or antihypertensive treatment, a plasma fasting triglyceride level of ≥150 mg/dL or lipid-lowering treatment, and a plasma HDL cholesterol level of ≤40 mg/dL in men or ≤50 mg/dL in women or lipid-lowering treatment. ALD was defined as the presence of SLD with alcohol consumption exceeding 350 g/week for women or 420 g/week for men, plus at least one cardiometabolic risk factor, or SLD with alcohol consumption exceeding 140 g/week for women or 210 g/week for men without cardiometabolic risk factors [15]. MetALD was defined as the presence of SLD, one or more cardiometabolic criteria, and alcohol consumption of 140–350 g/week for women or 210–420 g/week for men [15]. SLD with other etiologies was defined for individuals who met the definition of SLD but had a hepatitis C virus infection or another etiology [15].

### 2.3. Outcome Ascertainment

The outcomes included self-reported diagnoses of total CVD and its subtypes: stroke, congestive heart failure, and coronary heart disease. The participants were asked whether a healthcare provider had informed them of each CVD outcome before the survey. Additionally, all CVD cases were identified by self-reported use of anti-CVD drugs, which included medications for chronic rheumatic heart disease, ischemic heart disease, pulmonary heart disease, other forms of heart disease, cerebrovascular diseases, and diseases of the arteries, arterioles, and capillaries.

### 2.4. Sociodemographic Variables

The sociodemographic characteristics included age, sex, race/ethnicity, educational level, and ratio of family income to poverty. Age was categorized into three groups: 18–44 years, 45–64 years, and 65+ years. Race/ethnicity included Mexican American, non-Hispanic black, non-Hispanic white, other Hispanics, and others (Asian or Pacific Islander, Native American, and multiracial/-ethnic groups). The educational level was categorized as less than high school, high school or equivalent, or college or above. The ratio of family income to poverty was categorized as <1.0%, 1.0–2.9%, 3.0–4.9%, or ≥5.0% [16]. A higher income-to-poverty ratio indicates a higher family income status.

### 2.5. Metabolic Variables

The metabolic variables included obesity, hypertension, diabetes, and dyslipidemia. Obesity was defined as a body mass index of ≥30 kg/m^2^. Hypertension was characterized as a systolic blood pressure of ≥140 mmHg and/or diastolic blood pressure of ≥90 mmHg. Diabetes was identified based on fasting plasma glucose levels of ≥126 mg/dL or HbA1c levels of ≥6.5%. Dyslipidemia was defined as elevated total cholesterol (>200 mg/dL), low-density lipoprotein cholesterol (>100 mg/dL), or triglycerides (>150 mg/dL) and/or reduced high-density lipoprotein cholesterol (<40 mg/dL in men or <50 mg/dL in women).

### 2.6. Statistical Analysis

The analyses employed NHANES fasting subsample weights, considering nonresponse, noncoverage, and unequal probabilities of selection. The characteristics of participants in each survey cycle are presented as means and standard deviations (SDs) for continuous variables and numbers (percentages) for categorical variables.

We estimated the age-standardized prevalence and 95% confidence interval (CI) of MASLD in the US using the most recent NHANES 2017–2020 data and of total CVD and its subtypes among individuals with MASLD in each survey cycle. The prevalence rates were derived as the proportion of individuals with CVD (or its subtypes) relative to the total study population, with age standardization performed using all adults with MASLD as the standard population. The prevalence difference with a 95% CI between these two cycles and the related *p*-values were calculated using a two-sample z-test for proportions. We estimated the age-standardized prevalence and 95% CI of both total CVD and its subtypes among individuals with MASLD, overall and stratified by sociodemographic characteristics, namely, age, sex, race/ethnicity, educational level, and the ratio of family income to poverty. We also evaluated the prevalence ratio (PR) of CVD among individuals with MASLD versus those without the condition, in both the overall population and those with specific sociodemographic backgrounds. Poisson regression models were used to adjust for the sociodemographic and metabolic characteristics. All estimates and 95% CIs were weighted for national representation.

All analyses were performed using R software (version 4.0.3; Vienna, Austria). A two-tailed *p*-value of <0.05 was considered statistically significant.

## 3. Results

From 2017 to 2020, the weighted prevalence of MASLD was 31.2% (95% CI: 29.1%, 33.3%), corresponding to 61.9 million US adults out of the total 198.5 million. Table 1 summarizes the characteristics of individuals with and without MASLD. Individuals with MASLD had a mean age of 53 years, and 57% of them were men. The ethnic distribution included 12% Mexican American, 9% non-Hispanic black, 64% non-Hispanic white, 7% other Hispanics, and 8% other races/ethnicities. Compared to adults without MASLD, those with MASLD were older, predominantly male, and more likely to be of Mexican American ethnicity. They also had a lower proportion of individuals who had attended college or higher education. No significant differences were observed in the ratio of family income to poverty. Similar characteristics were observed in the NHANES III cohort (Table S1), where 24.7% (95% CI: 22.8%, 26.6%), or 33.1 million out of 134.0 million US adults, were living with MASLD.

### 3.1. Absolute Burden of CVD Among Individuals with MASLD

From 2017 to 2020, 17.0% (95% CI: 13.7%, 20.3%) of individuals with MASLD had comorbid CVD (Table 2). The age-standardized prevalence rates (95% confidence intervals [CIs]) of stroke, congestive heart failure, and coronary heart disease among individuals with MASLD were 4.1% (2.8%–5.4%), 3.9% (2.3%–5.5%), and 5.3% (3.3%–7.3%), respectively. The prevalence of all conditions significantly increased with age (*p* < 0.001), ranging from 3.3% (1.3%, 5.3%) to 35.5% (29.0%, 42.0%) for total CVD, from 0.9% (0.2%, 1.5%) to 9.6% (7.1%, 12.0%) for stroke, from 0.4% (−0.1%, 0.8%) to 5.6% (3.6%, 7.7%) for congestive heart failure, and from 0.7% (−0.3%, 1.7%) to 10.6% (7.2%, 13.9%) for coronary heart disease. Significant differences were observed across racial/ethnic groups (*p* < 0.05). Non-Hispanic whites had the highest prevalence of total CVD (21.6%) and coronary heart disease (7.3%), while non-Hispanic blacks had a notably high prevalence of stroke (8.0%) and congestive heart failure (5.9%). There were no significant differences in the prevalence rates across the sex and income groups.

**Figure 2:**
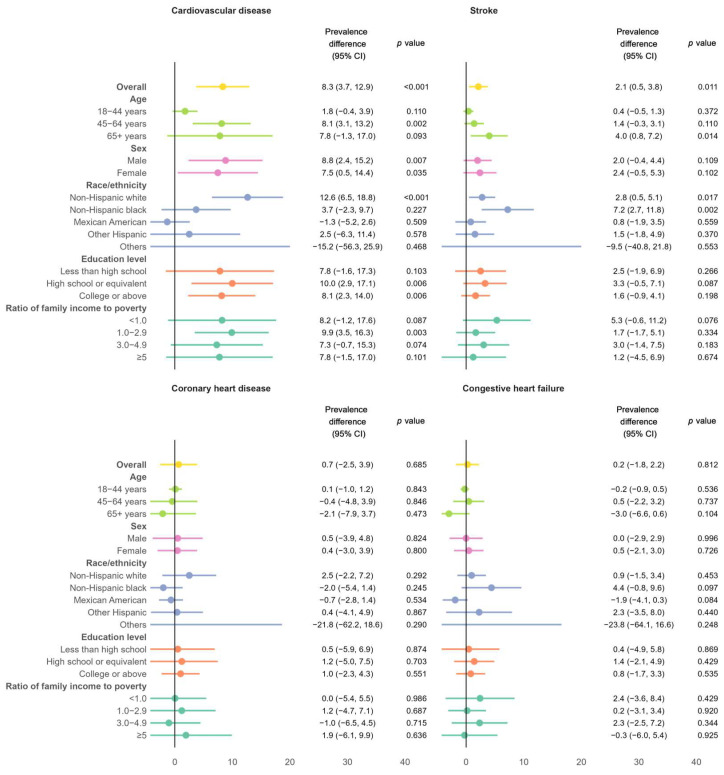
Differences in age-standardized prevalence of total CVD and its subtypes between the NHANES III cohort and the NHANES 2017–2020 cohort, overall and stratified by age, sex, race/ethnicity, education level, and ratio of family income to poverty.

Figure 2 illustrates the differences in the prevalence of various cardiovascular conditions between the NHANES III cohort and the NHANES 2017–2020 cohort. Over the past 30 years, the absolute age-standardized prevalence of total CVD increased by 8.3% (95% CI: 3.7%, 12.9%) and the stroke prevalence increased by 2.1% (0.5%, 3.8%). These increases were equivalent to 95% and 105% of the prevalence rates in the NHANES III cohort. The increase in total CVD was more pronounced in adults aged 45 years or older, non-Hispanic whites, individuals who attended high school, and those with a ratio of family income to poverty of 1.0–2.9. The increase in stroke prevalence was particularly notable among older adults, non-Hispanic whites, and non-Hispanic blacks. No significant changes were observed in the prevalence of congestive heart failure or coronary heart disease.

### 3.2. Relative Burden of CVD Among Individuals with MASLD Versus Those Without

**Figure 3:**
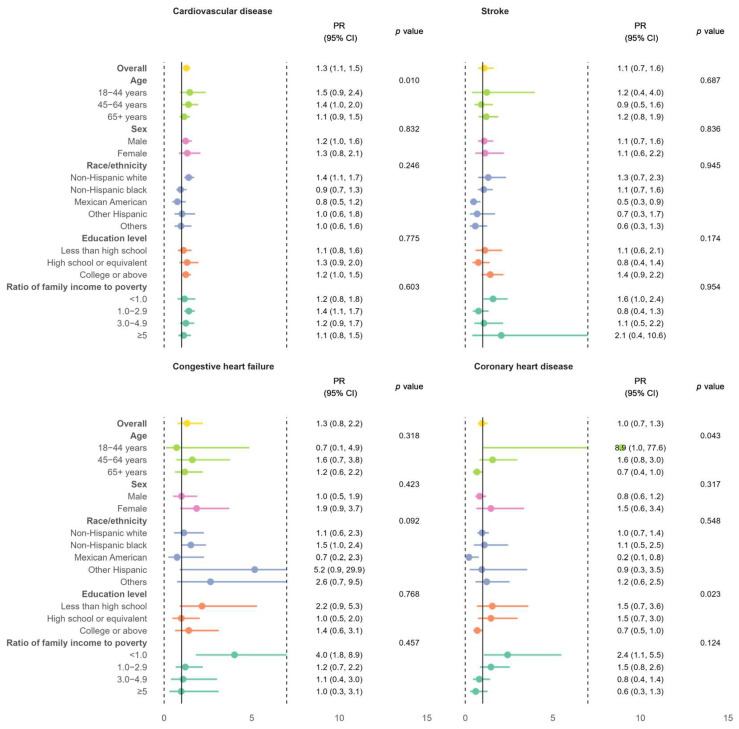
Adjusted prevalence ratios with 95% confidence intervals of total CVD and its subtypes among adults with MASLD compared to those without MASLD in NHANES 2017–2020, overall and stratified by age, sex, race/ethnicity, education level, and ratio of family income to poverty. The adjusted variables included all listed sociodemographic factors and metabolic factors including obesity, hypertension, diabetes, and dyslipidemia. CI: confidence interval; PR: prevalence ratio.

Compared to their counterparts without MASLD, MASLD individuals had a higher prevalence of total CVD after adjustments for socioeconomic and metabolic factors, with a fully adjusted PR (95% CI) of 1.3 (1.1, 1.5). The adjusted PRs for individuals with MASLD versus those without were more pronounced in younger age groups (interaction *p*-value of <0.05) (Figure 3).

## 4. Discussion

### 4.1. Principle Findings

To our knowledge, this is the first study to report the trends and disparities in CVD events among adults with MASLD in the US. Based on a nationally representative population with multiracial backgrounds, we report the most recent national estimates and trends of CVD and its subtypes, along with their sociodemographic disparities, among US adults with MASLD. In the period 2017–2020, the weighted prevalence of MASLD was 31.2%, affecting approximately 61.9 million US adults, of whom 17% were living with CVD. Comparing the NHANES III cohort to the NHANES 2017–2020 cohort showed an 8.3% increase in the absolute age-standardized prevalence of total CVD and a 2.1% increase in stroke prevalence over the past 30 years. These increases were particularly notable among older adults, non-Hispanic whites, and those with higher education and income. Furthermore, individuals with MASLD had a higher prevalence of total CVD than those without MASLD, even after adjustments for socioeconomic and metabolic factors. These relative differences were more pronounced in younger age groups.

### 4.2. Rising CVD Prevalence in Adults with MASLD

According to the US National Center for Health Statistics, there was a stable trend in the prevalence of heart disease in the general population aged ≥18 years from 2009 to 2019 [17]. Our observations in the population with MASLD showed a similar trend from 1988 to 2020. Analyses of NHANES data revealed stable stroke prevalence rates from 1999–2002 to 2015–2018 [18] and a decline in 10-year CVD risk, as estimated using the modified Framingham risk score and the American College of Cardiology and American Heart Association Atherosclerotic Cardiovascular Disease risk calculator, among CVD-free individuals with NAFLD from 1999 to 2016 [19]. However, our study found that the age-standardized stroke prevalence doubled and the total CVD prevalence increased by 95% in the MASLD population from 1988–1994 to 2017–2020. These contrasting trends highlight unique risk factors and dynamics in MASLD-related cardiovascular health, emphasizing the need to better understand the interplay between MASLD and CVD within broader population health trends.

The rising CVD prevalence over time among individuals with MASLD may be attributed to several temporal factors, including shifts in population demographics, evolving comorbidities, and advancements in detection and reporting. Aging populations [20] and the increasing prevalence of CVD risk factors (including obesity [21], hypertension [22], and diabetes [23]), both of which are strongly linked to MASLD, have likely contributed to the upward trend. Additionally, improvements in diagnostic techniques, such as the adoption of transient elastography in NHANES 2017–2020, which detects mild steatosis more reliably than historical ultrasound [24], may partially explain the observed trend. Changes in lifestyle factors, such as sedentary behavior [25] and dietary patterns [26], over the decades could also play a role.

### 4.3. Elevated CVD Risk Among Individuals with MASLD Versus Those Without

Not surprisingly, our study observed a higher prevalence of CVD in individuals with MASLD compared to those without, aligning with a robust body of prior evidence. Multiple large-cohort studies have consistently linked MASLD to increased CVD risk, including coronary heart disease, heart failure, and stroke [27,28]. Mendelian randomization analyses suggest that MASLD is causally associated with an elevated risk of arterial stiffness [29] but not with heart failure, stroke, coronary heart disease, or other CVD subtypes, indicating that metabolic factors like insulin resistance and chronic inflammation, which are hallmarks of MASLD, may play a central role in driving cardiovascular risk by promoting endothelial dysfunction, atherosclerosis, and other CVD-related pathways [30,31].

### 4.4. Sociodemographic Disparities

Our investigation revealed a rising prevalence of total CVD among older age groups among individuals with MASLD, aligning with well-established trends observed in the general population, both in the US and globally [32,33,34]. Furthermore, an investigation into CVD prevalence within the US general population revealed higher rates among non-Hispanic black and white individuals compared to Hispanics [35]. Previous studies also suggested an elevated risk of CVD among individuals with lower education levels [15,36] and those with lower income status [15,37]. Our analysis generalized these patterns to the prevalence of heart disease among subgroups of individuals with MASLD. Extending this evidence, our study further emphasized that the heightened prevalence of CVD associated with MASLD was even more pronounced among younger individuals, a pattern that is consistent with observations in the general population [35,38] and extending across various health domains [39,40]. It could be attributed to the earlier onset and potentially greater severity of metabolic dysfunction, such as insulin resistance, dyslipidemia, and systemic inflammation, in younger individuals with MASLD, amplifying their CVD risk [30,39]. In older populations, the presence of additional comorbidities or competing risk factors may dilute the relative contribution of MASLD to cardiovascular outcomes. These findings highlight MASLD as a potential early marker of heightened cardiovascular vulnerability in younger adults, underscoring the importance of early detection and targeted interventions in this demographic to mitigate long-term CVD risk.

### 4.5. Implications

These nuanced findings have significant implications for understanding the multifaceted interplay between MASLD and CVD across sociodemographic factors. Recognizing these disparities is crucial for developing targeted interventions and risk management strategies that consider the specific characteristics and challenges associated with different age groups, ethnicities, education levels, and income statuses. Moving forward, research should prioritize identifying optimal screening approaches and therapeutic strategies for high-risk subgroups. For example, younger individuals with MASLD may benefit from earlier CVD screening to prevent adverse outcomes, while revisions to treatment thresholds and updates to clinical guidelines could better address sociodemographic disparities, particularly among those facing socioeconomic or ethnic barriers to care. Policymakers might consider reorienting prevention frameworks toward equity by directing resources to underserved communities, exploring expanded Medicaid/Medicare support for metabolic health programs, and addressing structural factors like food insecurity or inequitable care access.

MASLD is a key component of metabolic disorders (diabetes, hypertension, hyperlipidemia) and cardiometabolic disease, according to the American Association of Clinical Endocrinology Clinical Practice Guideline [41,42]. Current recommendations, including those from the World Gastroenterology Organization, advise regular CVD risk monitoring every 1–2 years in MASLD patients and emphasize weight management and lifestyle modification as foundational interventions [41,42,43,44]. Lifestyle modifications include adopting a healthy diet (e.g., a Mediterranean or plant-based diet), increasing physical activity, and achieving weight loss through calorie restriction [41,42,43,44]. Pharmacological agents, such as GLP-1 receptor agonists, pioglitazone, and vitamin E, have shown promise in improving metabolic parameters and reducing liver fat in MASLD patients, though their use should be tailored to individual risk profiles and clinical guidelines [45]. A UK Biobank analysis (n = 78,000) underscored lifestyle risks and obesity as major contributors to MASLD complications [46], while NHANES data suggest that achieving all seven American Heart Association cardiovascular health metrics (e.g., smoking cessation, healthy diet, glucose control) could reduce all-cause and CVD deaths by 66% and 83%, respectively, among MASLD patients [47]. Our findings highlight the need for sociodemographically tailored health programs to optimize preventive strategies targeting MASLD-related risks. Future research should further explore the efficacy of combined lifestyle and pharmacological approaches in diverse populations.

### 4.6. Strengths and Limitations

The primary strength of our study lies in the utilization of NHANES data, allowing us to present multiethnic and nationally representative estimates for adults in the US with MASLD. However, this study has some limitations. First, the complex sampling design employed by the NHANES, while aiming for representativeness, may result in the underrepresentation of specific demographic groups [12,48]. This could limit the generalizability of the findings to the entire population, particularly for underrepresented subgroups such as certain racial or ethnic minorities or individuals in geographically isolated areas. Second, the absence of ultrasound data in the NHANES cycles from 1999 to 2016 made it impossible to evaluate the temporal trends of CVD prevalence among individuals with MASLD, further limiting the comprehensiveness of our analysis. Third, some of the data in the NHANES, such as alcohol consumption and subtypes of CVD, were self-reported, which could introduce recall bias and reporting inaccuracies. Specifically, the definition of MASLD excludes individuals with significant alcohol intake, but self-reported alcohol use is prone to underreporting, potentially leading to the misclassification of MASLD cases. This misclassification could skew the prevalence estimates and the observed associations between MASLD and CVD, thereby affecting the validity of the results. Fourth, liver biopsy is the gold standard for identifying steatosis and advanced hepatic fibrosis. However, due to the limitations of the NHANES study, we were only able to assess these conditions using noninvasive tests with as much specificity and sensitivity as possible. This could result in the misdiagnosis of some individuals, underestimating the burden of MASLD and its association with CVD. Fifth, the study is limited by the lack of longitudinal follow-up, which prevents the assessment of causality or long-term outcomes. Sixth, potential survival bias may exist, as individuals with severe MASLD or CVD may have died and, thus, are not represented in the NHANES 2017–2020 data. Finally, confounding by undiagnosed liver diseases, such as viral hepatitis or undetected cirrhosis, could influence the observed relationships between MASLD and CVD.

## 5. Conclusions

In summary, in two cross-sectional surveys weighted to be representative of the adult US population, the CVD prevalence among individuals with MASLD doubled from 1988–1994 to 2017–2020. Notably, sociodemographic disparities significantly influenced the burden of CVD associated with MASLD. This study calls for individualized screening, prevention strategies, and targeted policy decisions to effectively address these disparities and promote cardiovascular health.

## Figures and Tables

**Table 1:** Characteristics of US adults with and without MASLD in NHANES 2017–2020.

	With MASLD	Without MASLD	*p*-Value
No. of adults	2344	5088	
Age, years [mean (SD)]	53.1 (15.7)	49.3 (17.3)	<0.001
Age group, years (%)			<0.001
18–44 years	2179 (48.6)	721 (35.8)	
45–64 years	1729 (33.3)	1006 (41.6)	
65+ years	1180 (18.2)	617 (22.6)	
Sex (%)			
Male	1335 (60.0)	2364 (45.5)	<0.001
Female	1009 (43.0)	2724 (53.5)	
Race (%)			<0.001
Mexican American	392 (12.3)	466 (6.6)	
Non-Hispanic black	511 (8.7)	1508 (12.7)	
Non-Hispanic white	888 (63.5)	1690 (62.9)	
Other Hispanic	243 (7.0)	529 (7.9)	
Others ^a^	310 (8.4)	895 (9.9)	
Education (%)			0.019
Less than high school	413 (9.8)	910 (10.4)	
High school or equivalent	590 (30.0)	1219 (25.4)	
College or above	1338 (60.2)	2955 (64.2)	
Ratio of family income to poverty level (%)			0.367
<1.0	348 (10.9)	842 (12.6)	
1.0–2.9	903 (35.3)	1825 (32.6)	
3.0–4.9	427 (26.1)	868 (24.6)	
≥5.0	385 (27.6)	846 (30.2)	

NHANES weights were adjusted to generate nationally representative percentages. ^a^: Asian or Pacific Islander, Native American, and multiracial/-ethnic groups. MASLD, metabolic dysfunction-associated steatotic liver disease; NHANES, National Health and Nutrition Examination Survey; SD, standard deviation.

**Table 2:** Age-standardized prevalence (%) of total CVD and its subtypes among US adults with MASLD in NHANES 2017–2020, reported as rates (95% CIs).

	Total CVD	*p*-Value	Stroke	*p*-Value	Congestive Heart Failure	*p*-Value	Coronary Heart Disease	*p*-Value
Overall	17.0 (13.7, 20.3)		4.1 (2.8, 5.4)		3.9 (2.3, 5.5)		5.3 (3.3, 7.3)	
Age		<0.001		<0.001		<0.001		<0.001
18–44 years	3.3 (1.3, 5.3)		0.9 (0.2, 1.5)		0.4 (−0.1, 0.8)		0.7 (−0.3, 1.7)	
45–64 years	18.7 (14.3, 23.1)		3.9 (2.6, 5.1)		4.3 (2.1, 6.4)		6.3 (2.4, 10.2)	
65+ years	35.5 (29.0, 42.0)		9.6 (7.1, 12.0)		5.6 (3.6, 7.7)		10.6 (7.2, 13.9)	
Sex		0.462		0.464		0.720		0.065
Male	18.3 (13.3, 23.3)		3.7 (2.0, 5.4)		4.2 (2.2, 6.2)		6.7 (4.0, 9.4)	
Female	15.3 (9.8, 20.8)		5.5 (3.0, 7.9)		3.5 (1.4, 5.7)		3.3 (0.8, 5.8)	
Race/ethnicity		<0.001		0.001		0.025		<0.001
Non-Hispanic white	21.6 (16.8, 26.3)		4.8 (2.9, 6.7)		4.3 (2.0, 6.7)		7.3 (4.4, 10.3)	
Non-Hispanic black	13.6 (8.7, 18.4)		8.0 (4.4, 11.6)		5.9 (2.5, 9.3)		2.3 (0.1, 4.5)	
Mexican American	6.8 (2.5, 11.1)		1.3 (−0.7, 3.3)		2.2 (−0.4, 4.7)		3.3 (0.4, 6.2)	
Other Hispanic	12.1 (4.9, 19.2)		1.9 (−0.4, 4.1)		3.2 (−0.4, 6.8)		2.6 (0.8, 4.4)	
Others	14.3 (5.4, 23.3)		4.1 (−0.3, 8.5)		2.7 (−0.8, 6.2)		3.0 (0.3, 5.6)	
Education level		0.294		0.130		0.056		0.357
Less than high school	21.1 (13.5, 28.7)		5.8 (2.3, 9.3)		6.7 (2.9, 10.6)		7.4 (2.6, 12.1)	
High school or equivalent	22.7 (14.6, 30.8)		6.5 (2.2, 10.8)		3.3 (1.3, 5.2)		7.4 (1.7, 13.1)	
College or above	15.7 (11.9, 19.5)		3.3 (1.9, 4.8)		3.7 (1.5, 5.9)		4.6 (2.8, 6.3)	
Ratio of family income to poverty		0.149		0.259		0.176		0.430
<1.0	17.0 (9.3, 24.6)		9.8 (3.8, 15.8)		8.8 (3.0, 14.5)		5.2 (1.1, 9.2)	
1.0–2.9	20.4 (15.1, 25.6)		5.5 (2.1, 9.0)		4.6 (2.3, 6.9)		6.6 (2.4, 10.8)	
3.0–4.9	16.8 (10.0, 23.5)		3.6 (1.0, 6.3)		3.2 (−0.3, 6.6)		4.5 (1.7, 7.3)	
≥5	17.4 (10.8, 23.9)		6.6 (2.2, 10.9)		2.8 (0.4, 5.1)		10.1 (4.1, 16.2)	

## Data Availability

The data used in this study are publicly available from https://wwwn.cdc.gov/nchs/nhanes/Default.aspx (accessed on 20 November 2023).

## Supplementary Materials

The following supporting information can be downloaded at https://www.mdpi.com/article/10.3390/biomedicines13040956/s1, Table S1: Characteristics of US adults with and without MASLD in NHANES III cohort (1988–1994).

## Abbreviations

The following abbreviations are used in this manuscript:

CI	Confidence interval
CVD	Cardiovascular disease
MASLD	Metabolic dysfunction-associated steatotic liver disease
NAFLD	Non-alcoholic fatty liver disease
NHANES	National Health and Nutrition Examination Survey
PR	Prevalence ratio
