# Supplementary material for: Trends and Disparities in Cardiovascular Disease in US Adults with Metabolic Dysfunction-Associated Steatotic Liver Disease
Source: Biomedicines. 2025 Apr 13;13(4):956. doi: 10.3390/biomedicines13040956 (PMC12024783; doi:10.3390/biomedicines13040956)
Supplement: Supplementary file 1 [file biomedicines-13-00956-s001.zip › biomedicines-3494268-supplementary.pdf]

**Table S1.** Characteristics of US adults with and without MASLD in NHANES III cohort (1988–1994).

| | **With MASLD** | **Without MASLD** | ***p* Value** |
|---|---|---|---|
| No. of adults | 3589 | 9559 | |
| Age, year [mean (SD)] | 47.9 (14.6) | 42.0 (14.5) | <0.001 |
| Age group, year (%) | | | <0.001 |
| 18–44 years | 5923 (66.1) | 1614 (48.9) | |
| 45–64 years | 2387 (25.1) | 1304 (36.5) | |
| 65+ years | 1249 (8.8) | 671 (14.6) | |
| Male (%) | 1655 (51.2) | 4425 (46.8) | 0.008 |
| Race (%) | | | <0.001 |
| Mexican American | 1370 (7.4) | 2495 (4.9) | |
| Non–Hispanic Black | 768 (8.5) | 3074 (12.0) | |
| Non–Hispanic White | 1330 (76.4) | 3579 (75.6) | |
| Other Hispanic | 84 (4.6) | 258 (3.9) | |
| Others [a] | 37 (3.0) | 153 (3.6) | |
| Education (%) | | | <0.001 |
| Less than high school | 1003 (12.9) | 1764 (8.7) | |
| High school of equivalent | 1688 (51.2) | 4684 (46.4) | |
| College or above | 880 (35.9) | 3049 (44.9) | |
| Ratio of family income to poverty level (%) | | | 0.232 |
| <1.0 | 821 (12.9) | 1988 (12.4) | |
| 1.0–2.9 | 1566 (44.2) | 3969 (40.7) | |
| 3.0–4.9 | 605 (27.1) | 1878 (30.1) | |
| ≥5.0 | 286 (15.7) | 909 (16.8) | |

NHANES survey weights were adjusted to generate nationally representative percentages. [a]: Including Asian or Pacific Islander, Native American, and multiracial/ethnic groups. MASLD: metabolic dysfunction-associated steatotic liver disease; NHANES: National Health and Nutrition Examination Survey; SD: standard deviation.
